# Serum Neurofilaments in Motor Neuron Disease and Their Utility in Differentiating ALS, PMA and PLS

**DOI:** 10.3390/life13061301

**Published:** 2023-05-31

**Authors:** Gavin McCluskey, Karen E. Morrison, Colette Donaghy, John McConville, Mark O. McCarron, Ferghal McVerry, William Duddy, Stephanie Duguez

**Affiliations:** 1 Personalised Medicine Centre, School of Medicine, Ulster University, Derry BT47 6SB, UK; mccluskey-g@ulster.ac.uk (G.M.); w.duddy@ulster.ac.uk (W.D.); 2 Department of Neurology, Royal Victoria Hospital, Belfast BT12 6BA, UK; k.morrison@qub.ac.uk (K.E.M.); john.mcconville@setrust.hscni.net (J.M.); 3 Department of Neurology, Altnagelvin Hospital, Derry BT47 6SB, UK; coletteg.donaghy@westerntrust.hscni.net (C.D.); mark.mccarron@westerntrust.hscni.net (M.O.M.); ferghal.mcverry@westerntrust.hscni.net (F.M.); 4 Faculty of Medicine, Health & Life Sciences, Queen’s University, Belfast BT9 6AG, UK; 5 Department of Neurology, Ulster Hospital, Belfast BT16 1RH, UK

**Keywords:** motor neuron disease, amyotrophic lateral sclerosis, progressive muscular atrophy, primary lateral sclerosis, neurofilament light, neurofilament heavy

## Abstract

Neurofilament levels are elevated in many neurodegenerative diseases and have shown promise as diagnostic and prognostic biomarkers in Amyotrophic Lateral Sclerosis (ALS), the most common form of Motor Neuron Disease (MND). This study assesses serum neurofilament light (NFL) and neurofilament heavy (NFH) chain concentrations in patients with ALS, other variants of motor neuron disease such as Progressive Muscular Atrophy (PMA) and Primary Lateral Sclerosis (PLS), and a range of other neurological diseases. It aims to evaluate the use of NFL and NFH to differentiate these conditions and for the prognosis of MND disease progression. NFL and NFH levels were quantified using electrochemiluminescence immunoassays (ECLIA). Both were elevated in 47 patients with MND compared to 34 patients with other neurological diseases and 33 healthy controls. NFL was able to differentiate patients with MND from the other groups with a Receiver Operating Characteristic (ROC) curve area under the curve (AUC) of 0.90 (*p* < 0.001). NFL correlated with the rate of disease progression in MND (rho 0.758, *p* < 0.001) and with the ALS Functional Rating Scale (rho −0.335, *p* = 0.021). NFL levels were higher in patients with ALS compared to both PMA (*p* = 0.032) and PLS (*p* = 0.012) and were able to distinguish ALS from both PMA and PLS with a ROC curve AUC of 0.767 (*p* = 0.005). These findings support the use of serum NFL to help diagnose and differentiate types of MND, in addition to providing prognostic information to patients and their families.

## 1. Introduction

Motor Neuron Disease (MND) is a progressive neurodegenerative disease resulting in progressive muscular weakness, bulbar dysfunction and respiratory impairment, with a typical survival of 2–4 years from disease onset [1]. The most common form of MND, Amyotrophic Lateral Sclerosis (ALS), involves both upper and lower motor neuron dysfunction, while Progressive Muscular Atrophy (PMA) and Primary Lateral Sclerosis (PLS) are variants of MND with clinical features restricted to dysfunction of solely lower or upper motor neurons, respectively [2]. While it is recognized that PMA and PLS share many pathological features with ALS and there is ongoing discussion regarding whether they, with ALS, should be considered as a single disease spectrum [3,4,5], both these subtypes have much slower rates of progression and longer survival than ALS, and thus distinguishing them from ALS is of prognostic importance [4,6,7]. There is difficulty in confidently diagnosing these different phenotypes based on a single assessment, as clinical signs may, and do, change with time. It is recommended that a PLS diagnosis be given only after evidence of the absence of lower motor neuron signs has been established 4 years after symptom onset [8]. There has been extensive research over the last 20 years to develop biomarkers in ALS that could improve diagnostic accuracy and reduce the typical 12-month delay to diagnosis [9]. In addition, biomarkers are needed to help predict the rate of disease progression and help with patient stratification in clinical trials [10]. The recent Airlie House guidelines have recommended that biomarkers be included as a best practice for clinical trial design in ALS [11].

Neurofilaments (NFs) have emerged as promising diagnostic and prognostic biomarkers in MND [12]. NFs are type IV intermediate filaments and form a major cytoskeletal protein specifically expressed in neurons [13]. They are typically separated by their molecular weight into light (NFL), medium (NFM) and heavy (NFH) chains. They are released into the cerebrospinal fluid (CSF) after neuronal damage [13]. Multiple methods have been used to quantify these proteins. Initially, enzyme-linked immunosorbent assays (ELISAs) were developed to detect NFL and phosphorylated NFH (pNFH) in CSF. However, because of their limited sensitivity, they are not recommended to measure the typically 50–100-fold lower concentrations of NFs in the blood [14]. More recently, electrochemiluminescence immunoassays (ECLIA) and Single-Molecule Array (Simoa) assays have been developed [15,16]. While the Simoa assay is the most sensitive method of detection, both ECLIA and Simoa can reliably detect NF levels in blood samples and show a correlation with CSF levels, with a recent meta-analysis showing a good correlation between CSF and blood (plasma or serum) NF levels with r = 0.69 and 0.68 for ECLIA and Simoa methods, respectively [14]. Serum NFL levels measured by ECLIA and Simoa have also been shown to correlate strongly with each other (r = 0.86) [15].

NFL and NFH levels in the serum, plasma and CSF are abnormal in a wide range of neurological diseases [17,18]. Studies have shown that both NFL and NFH levels are highly elevated in ALS and correlate with measures of disease progression in ALS [19,20,21], leading to measures of NFL and NFH now being incorporated into clinical trial designs in ALS as a secondary outcome measure [22,23]. While there has been a large body of work evaluating NFL and NFH in ALS, there has been much less study in PMA and PLS. Some studies have shown higher levels of NFL in ALS compared to both PLS and PMA [24], although other studies have not shown significant changes between all groups [25,26,27]. This may be due to the often small numbers of PMA and PLS in these studies, reflective of the fact that these are rarer diseases and each often represent less than 5% of MND cohorts [7,28].

This study evaluates serum NFL and NFH in MND compared to other neurodegenerative and neuromuscular diseases. We also evaluate the prognostic utility of NFL and NFH in MND compared to clinical prognostic tools and, finally, determine the utility of serum NFL and NFH in differentiating ALS from PMA and PLS.

## 2. Materials and Methods

### 2.1. Ethical Approval

All samples used in this study were obtained from the Northern Ireland Motor Neuron Disease Biobank. The NI MND biobank has received ethical approval from the Health and Social Care Research Ethics Committee A (REC reference: 21/NI/0010). This study has been carried out following the conditions of ethical approval for the use of these samples.

### 2.2. Sample Collection

Blood samples from patients were collected in serum separator tubes. After a minimum of 30 min, samples were centrifuged at 1800× *g* for 15 min at 4 °C. The serum was then aliquoted into sterile microtubes and stored at −80 °C in the biobank until use.

### 2.3. Participants and Clinical Parameters

All patients were aged 18 years and over. Patients with MND (ALS, PMA and PLS) were diagnosed by experienced MND specialist neurologists according to the revised El-Escorial criteria. Patients were classified as PMA or PLS if there were pure lower or motor neuron signs, respectively. PLS was only confirmed in patients who had only upper motor neuron signs present for at least 4 years with no evidence of lower motor neuron involvement on electrophysiological studies (nerve conduction studies and electromyography) according to 2020 consensus diagnostic criteria [8]. Patients had revised ALS Functional Rating Scores (ALSFRS-r) and King’s staging scores taken on the same day as sample collection [29,30]. The rate of change in ALSFRS-r (DeltaFS) was used to determine the rate of disease progression. This was calculated as (48-ALSFRS-r score)/disease duration in months from symptom onset at assessment [31]. Familial MND was defined as having a known first- or second-degree relative diagnosed with MND or a first-degree relative diagnosed with Frontotemporal Dementia (FTD). All patients in this study that were identified as ‘neuromuscular disease controls’ had a diagnostic evaluation by experienced neurologists and had genetic confirmation of diagnosis where appropriate, e.g., patients with Spinal and Bulbar Muscular Atrophy (SBMA) and Spinal Muscular Atrophy (SMA).

### 2.4. NFL and NFH Quantification

Serum NFL and NFH concentrations were quantified by ECLIA using commercial kits (K1517XR-2 for NFL and K1517YR-2 for NFH) provided by Meso Scale Diagnostics (MSD), Rockville, Maryland, USA. The assay was performed according to the manufacturer’s instructions. In brief, 25 μL of the relevant biotinylated antibody prepared in MSD Diluent 100 was added to the MSD GOLD 96-well Small Spot Streptavidin plates and incubated at 20 °C for 1 h on a rotary shaker at 700 rpm. The plate was then washed three times with 200 μL PBS (Oxoid, Basingstoke, UK) supplemented with Tween-20 (Merck, Darmstadt, Germany) 0.05% (*v*/*v*) (PBS-T). After adding 25 μL of MSD Diluent 12 and 25 μL of NF standard calibrators, the 50% diluted serum samples were plated in duplicate and incubated at 20 °C with shaking for 1 h. After three washes with PBS-T, 50 μL of the SULFO-TAG™-conjugated detection antibody diluted in MSD Diluent 11 was plated and incubated at 20 °C with shaking for 1 h. The plates were washed further three times, and then 150 μL of MSD GOLD Read Buffer B was added, and the plates were read immediately on the MESO QuickPlex SQ 120MM instrument. The sample concentrations were calculated using the MSD discovery workbench 4.0 analysis software. Three samples were analyzed on all plates as internal controls to assess inter-assay variability. The mean intra-assay coefficient of variation (CV) was 6.4% for the NFH assays and 6.0% for the NFL assays. The mean inter-assay CV was 19.0% for the NFH assays and 6.7% for the NFL assays.

### 2.5. Statistical Methods

Data were analyzed using SPSS v.26 (IBM, Armonk, NY, USA) and Prism v.9 (GraphPad Software, San Diego, CA, USA). The Shapiro–Wilk test was used to assess for normality and Levene’s test of homogeneity of variance was used to test for heteroscedasticity, and parametric or non-parametric tests were used accordingly. The Shapiro–Wilk test showed NFL and NFH were skewed for some disease groups; however, there was significant heteroscedasticity between groups (Levene’s test *p* < 0.001 for both NFL and NFH), and therefore the Welch ANOVA was used with Dunnett’s T3 post-hoc test rather than the Kruskal–Wallis test to compare NFL and NFH between groups [32]. The Receiving Operating Characteristic (ROC) curves were created to evaluate the overall performance of the NFL and NFH to distinguish between groups. The Chi-squared test was used to compare categorical variables (e.g., gender, site of onset). The ALSFRS-r (*p* = 0.043) and DeltaFS (*p* < 0.001) were both non-normally distributed, and therefore the Spearman’s rank test was used for correlation of the NFL and NFH.

## 3. Results

### 3.1. Patient Characteristics

A total of 114 patients were included in the study. Table 1 shows the characteristics of all the included patients. There was no difference in gender between groups (*p* = 0.085). The patients with MND had a median age of 65 years at sample collection, which was older than both the disease controls (56 years) and healthy controls (59 years), *p* < 0.001. There were 47 patients with MND, comprising 34 with ALS, 8 with PMA and 5 with PLS. The disease-specific characteristics of patients with MND at the time of sampling are shown in Table 2. There was no difference in age between patients with ALS, PMA or PLS (*p* = 0.683). All of the patients with PMA were males, compared to 52.9% of those with ALS and 40% of those with PLS (*p* = 0.033). There was no difference in the site of onset between groups. No patients with PMA or PLS had a family history of MND or FTD compared with 23.5% of patients with ALS, although this difference was not statistically significant (*p* = 0.158). PLS is generally considered to be a sporadic disorder, with very few familial cases reported in the literature [8,33,34]. PMA has also historically been considered a sporadic disorder, although some ALS-associated genes have been reported in cases of PMA [33,35,36,37].

As would be expected, patients with PLS had a longer disease duration, with a median time from symptom onset to sampling of 175 months, compared to 58 months for patients with PMA and 26.5 months for patients with ALS (*p* = 0.008). There was no difference in the median ALSFRS-r scores between groups (*p* = 0.935). There was also no difference in the King’s stage scores between groups (*p* = 0.069), although the rate of disease progression measured by the DeltaFS was much slower for patients with PLS at 0.08 points per month compared to 0.19 for PMA and 0.47 for ALS (*p* < 0.001). The breakdown of the number of patients receiving riluzole or having clinical interventions such as gastrostomy or ventilatory support is also shown in Table 2.

### 3.2. Neurofilament Concentrations between Groups

**Figure 1:**
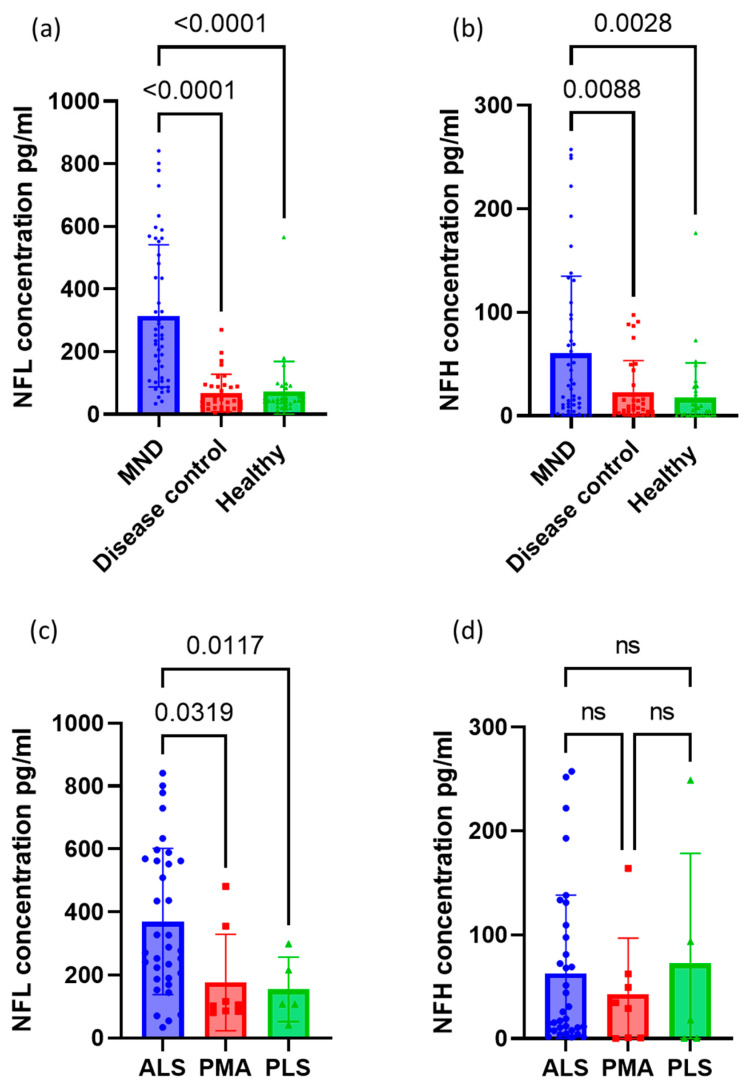
Column graphs showing the distribution of (**a**) NFL and (**b**) NFH concentration between MNDs, disease controls and healthy controls. Also shown is the distribution of (**c**) NFL and (**d**) NFH concentration between the different types of MND. Error bars represent the mean with standard deviation.

The serum NFL and NFH concentrations for each group are shown in Table 3. This is also shown in Figure 1. The mean NFL concentration was much higher in those with MND overall, at 314.0 pg/mL compared to 67.7 pg/mL for disease controls and 73.0 pg/mL for healthy controls (*p* < 0.001 for both). Serum NFH was also higher in patients with MND at 60.3 pg/mL, compared to 22.9 pg/mL for disease controls (*p* = 0.009) and 17.4 pg/mL for healthy controls (*p* = 0.003). For patients with MND, the mean serum NFL was highest for those with ALS at 369.9 pg/mL, compared with 176.5 pg/mL for PMA (*p* = 0.032) and 154.5 pg/mL for PLS (*p* = 0.012). There was no difference in mean NFH levels between ALS, PMA and PLS (*p* = 0.683). There was no difference in mean NFL or NFH levels in patients with the bulbar or spinal-onset disease (*p* = 0.402 and *p* = 0.181, respectively). There was no difference in familial vs. sporadic cases of MND for either NFL (286.3 vs. 319.7 pg/mL, *p* = 0.677) or NFH (83.9 vs. 55.4 pg/mL, *p* = 0.449).

Of the disease controls, MG had the lowest NFL and NFH values, although there were only two MG patients included. As shown in Table 3, SMA and SBMA, which are both inherited motor neuronopathies affecting lower motor neurons, had lower values of both NFL and NFH than PMA and other neuropathies. The overall ANOVA for NFL between these groups was statistically significant (*p* = 0.027); however, the post-hoc pairwise comparisons did not show any significant differences between groups (all *p* ≥ 0.1). There was no difference in serum NFH concentration between these lower motor neuron disorders (*p* = 0.073).

### 3.3. Receiver Operating Characteristics for Neurofilaments

**Figure 2:**
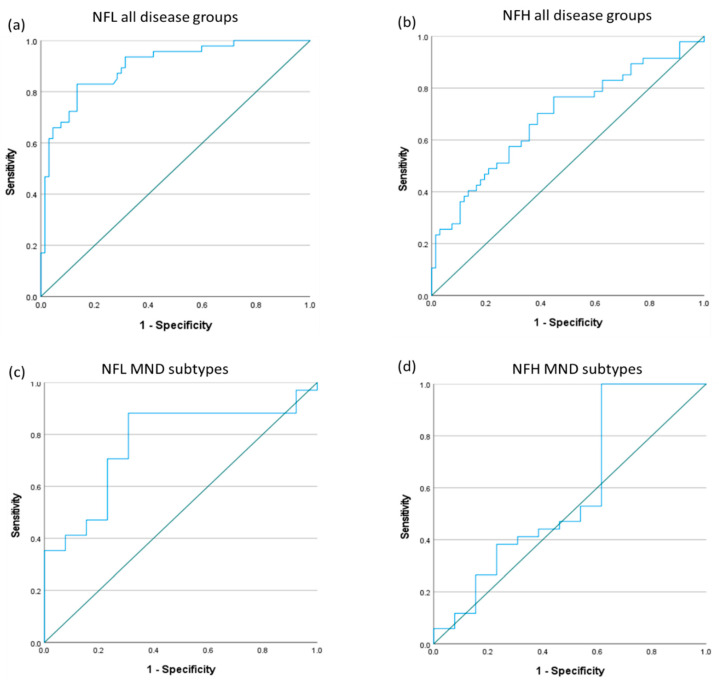
Receiver operating characteristic (ROC) curves showing the utility of (**a**) NFL and (**b**) NFH to correctly classify any MND from the disease and healthy controls. The bottom ROC curves show the utility of (**c**) NFL and (**d**) NFH to correctly classify ALS from PMA and PLS.

ROC curves were calculated to evaluate the overall ability of serum NFL and NFH levels to (a) identify patients with MND from other neurological diseases and healthy controls, and (b) identify patients with ALS from those with PMA and PLS. The ROC curves are shown in Figure 2.

The ROC curve for NFL had an area under the curve (AUC) of 0.90 (Figure 2a), showing an excellent ability to classify MND from other neurological diseases and healthy controls (*p* < 0.0001) [38]. A serum NFL cutoff level of ≥100.3 pg/mL gave a sensitivity of 83.0% and a specificity of 86.6% for identifying patients with MND. The AUC for NFH was much lower at 0.686 (Figure 2b), showing only a fair ability to classify MND (*p* = 0.001). There was a broad range of NFH levels in patients with MND, as shown in Figure 1, with many having a very low concentration, and therefore it had low sensitivity for detecting patients with MND. A serum NFH cutoff level of ≥34.0 pg/mL had a sensitivity of 44.7% and a specificity of 82.1%.

For patients with MND, the AUC for identifying patients with ALS from PMA and PLS was 0.767 (Figure 2c; *p* = 0.005) for NFL, showing moderate discriminatory value. The optimal cutoff in NFL concentration was 130.3 pg/mL, giving a sensitivity of 88.2% and specificity of 69.2%. The AUC for identifying patients with ALS from those with PMA and PLS using the serum NFH concentration was 0.590 (Figure 2d; *p* = 0.341), therefore showing poor discriminatory value.

### 3.4. Neurofilaments for Monitoring Disease Progression in MND

**Figure 3:**
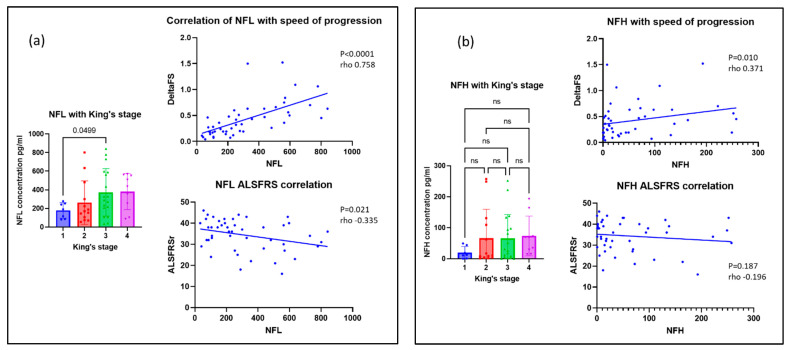
Scatter graphs showing the correlation of (**a**) NFL and (**b**) NFH with markers of disease severity and progression in MND.

Serum NFL and NFH concentrations were evaluated against measures of disease severity, including the ALSFRS-r and King’s staging score of patients at the time of sample collection. The correlation with deltaFS was also evaluated as a marker of the rate of disease progression. NFL levels were inversely correlated with ALSFRS-r (Spearman’s rho = −0.335, *p* = 0.021). NFL was strongly correlated with deltaFS (rho = 0.758, *p* < 0.001), and therefore higher NFL levels signified more rapid disease progression. NFL also correlated with King’s stage of disease (rho 0.328, *p* = 0.025). Welch’s ANOVA was significant for NFL with King’s stage (*p* = 0.016), although the only significant difference in post-hoc testing was the mean NFL for patients with stage 1 disease compared with stage 3 (*p* = 0.049). NFH was also correlated with deltaFS (rho = 0.371, *p* = 0.010). There was no correlation with ALSFRS-r or with King’s stage (*p* = 0.187 and 0.051, respectively). These data are shown in Figure 3.

## 4. Discussion

This study adds to the evidence that serum NFL and NFH concentrations can be used as non-invasive biomarkers in MND. Both NFL and NFH were raised in patients with MND compared to disease and healthy controls. NFL in particular showed strong diagnostic performance, with an AUC of 0.90 on the ROC curve. A recent review of studies evaluating the diagnostic use of NFL in MND compared to a variety of neurological conditions and healthy controls found the AUC for NFL measured in serum, plasma or CSF typically ranging from 0.81 to 0.97, showing good diagnostic utility [19]. A single study comparing NFL levels in ALS with acute and chronic inflammatory demyelinating polyneuropathies had a much lower AUC of only 0.58 [19]. In the present study, a serum NFL cutoff level of ≥100.3 pg/mL gave a sensitivity of 83.0% and a specificity of 86.6% for a diagnosis of MND. To fully assess the diagnostic utility of NFL, future studies will need to carefully define the target population (e.g., primary versus secondary care setting and which other conditions are to be excluded) and establish positive and negative predictive values applicable to the clinical setting [39]. There have been calls for increased international collaboration to further develop standardized NFL reference ranges and to determine the cut-off values for different clinical contexts, such as diagnosis or monitoring treatment response [40].

NFL also showed a very strong correlation with the deltaFS, further validating its use as a biomarker for tracking the rate of disease progression. Multiple studies have previously validated the use of serum NFL in ALS as a prognostic biomarker [19,20,21,41]. There has been much less research on the rare variants of MND, PMA and PLS, with some studies showing conflicting results on whether NFL levels in patients with ALS are higher than those evident in PMA and PLS [25,26,27]. In this study, serum NFL was able to distinguish ALS from PMA and PLS, with the ROC curve showing an AUC of 0.767. This is of importance clinically, as both PMA and PLS have much slower disease progression and longer survival [4,6,7]. The addition of serum NFL and NFH as clinical tests could aid in giving more personalized prognostic information to patients with MND. Typically, patients with MND are told that life expectancy is usually 2–4 years from symptom onset, although this is highly variable [1]. More personalized prognostic information, such as the ENCALS survival prediction model, has been shown to help provide patients with a sense of control and facilitate future care planning [42,43].

It is of interest that we did not find elevated serum NF levels in patients with SMA or SBMA, both of which disorders are due to lower motor neuron degeneration. Previous studies have shown that both NFL and NFH levels are elevated in infants with SMA but those levels decline over time [44,45]. This is in contrast to MND, where NF levels remain stable throughout the disease [46,47]. Six of the seven patients with SMA were on treatment with risdiplam, a small-molecule splicing modifier of the survival of motor neuron (SMN) 2-directed RNA. Previous studies in adults with SMA have found no difference in either NFL or NFH in patients pre- or post-treatment with nusinersen, an antisense oligonucleotide that alters SMN2 pre-mRNA splicing [48]. The NFL and NFH levels were not elevated pre- or post-treatment compared with healthy controls in that cohort [48]. Previous studies have also found that NFL and NFH were not elevated in patients with SBMA [49,50]. A small study of five patients with SBMA did report elevated plasma NFL [51]. It has been proposed that lower NF levels in SBMA could be due to the much slower progression seen in SBMA compared to MND [50]. However, this slow progression could not account for the elevations of NFL and NFH that have been reported in other slowly progressive neuropathies [52,53]. In our study, there was no statistically significant difference in NFL (*p* = 0.788) or NFH (*p* = 0.213) in the six patients with other forms of neuropathy compared to healthy controls. While SMA and SBMA can both be confirmed by genetic diagnosis, there is often a considerable delay before obtaining genetic results, and it has been suggested that including NFL and NFH levels as part of an initial biochemical screen could help diagnose patients being evaluated with motor neuron deficits [49].

The limitations of this study are the small numbers involved, particularly in the rare disease subtypes of PMA and PLS. There is much less published information on NF levels in these rare conditions; our data here adds to the body of published data and suggests that further validation of serum NF levels in larger numbers in these cohorts will be important to determine their clinical utility.

## 5. Conclusions

This study adds further validation to the use of serum NF levels, particularly NFL, as non-invasive biomarkers to aid in the diagnosis of MND, although a complete assessment of diagnostic utility will depend on the clinical context, such as the definition of the target population and consideration of adequate predictive values for that population. We also show that NFL levels correlate with the rate of progression of the disease and with disease severity. NFL levels in our study were also able to accurately differentiate patients with ALS from those with PMA and PLS.

## Figures and Tables

**Table 1:** Clinical characteristics of all included patients.

		MND	Disease Controls	Healthy Controls	*p* Value
Number of patients		47	34	33	
Gender	Male (%)	28 (59.6)	22 (64.7)	13 (39.4)	0.085
	Female (%)	19 (40.4)	12 (35.3)	20 (60.6)	
Median age at sampling years (IQR)		65 (58, 75)	56 (40.8, 66.3)	59 (51, 68.5)	<0.001
Disease subcategories		34 ALS	8 Myopathies		
		8 PMA	7 SMA		
		5 PLS	6 Neuropathies		
			6 PD		
			5 SBMA		
			2 MG		

ALS—amyotrophic lateral sclerosis, PMA—progressive muscular atrophy, PLS—primary lateral sclerosis, SMA—spinal muscular atrophy, SBMA—spinal bulbar muscular atrophy, PD—Parkinson’s disease, MG—myasthenia gravis.

**Table 2:** Clinical characteristics of patients with MND.

			ALS	PMA	PLS	*p* Value
Number			34	8	5	
Median age at sampling years(IQR)			64 (56.8, 75.3)	68 (64,74.3)	67 (57.5, 78)	0.683
Gender	Male (%)		18 (52.9)	8 (100)	2 (40)	0.033
	Female (%)		16 (47.1)	0 (0)	3 (60)	
Site of onset	Bulbar (%)		8 (23.5)	1 (12.5)	3 (60)	0.142
	Spinal (%)		26 (76.5)	7 (87.5)	2 (40)	
Familial (%)			8 (23.5)	0 (0)	0 (0)	0.158
Median disease duration months			26.5 (18.8, 45)	58 (28.5, 103)	175 (109, 213)	0.008
Median ALSFRS-r			37 (28.8, 40.3)	35 (26, 41.8)	34 (31, 36.5)	0.935
Median DeltaFS			0.47 (0.25, 0.64)	0.19 (0.17, 0.28)	0.08 (0.07, 0.14)	<0.001
King’s staging		Stage 1	4	3	0	0.069
		Stage 2	10	2	1	
		Stage 3	14	0	4	
		Stage 4	6	3	0	
Riluzole use (%)			30 (88.2)	6 (75)	2 (40)	0.034
Gastrostomy (%)			7 (20.6)	1 (12.5)	0 (0)	0.485
Non-invasive ventilation (%)			1 (2.9)	3 (37.5)	0 (0)	0.005
Tracheostomy (%)			0 (0)	0 (0)	0 (0)	

**Table 3:** Neurofilament concentrations for each disease category.

Disease		Number of Patients	NFL (pg/mL)	NFH (pg/mL)
			Mean (SD)	Mean (SD)
All MND		47	314.0 (227.1)	60.3 (74.7)
Type of MND	ALS	34	369.9 (232.4)	62.6 (75.4)
	PMA	8	176.5 (153.5)	42.6 (54.4)
	PLS	5	154.5 (102.5)	72.3 (105.9)
All Disease Controls		34	67.7 (59.9)	22.9 (30.5)
Disease controls	Neuropathy	6	79.9 (46.9)	39.1 (35.8)
	Myopathy	8	106.6 (59.9)	35.1 (38.9)
	MG	2	18.8 (1.7)	1.0 (1.2)
	PD	6	102.7 (83.0)	26.3 (32.9)
	SBMA	5	29.4 (7.6)	4.3 (2.7)
	SMA	7	23.9 (16.8)	11.9 (17.6)
Healthy Controls		33	73.0 (95.6)	17.4 (33.6)

## Data Availability

The data presented in this study are available on request from the corresponding author.
